# Perioperative Challenges in Oral Cavity Cancer Reconstruction in a Patient with Behçet’s Disease: A Case Report

**DOI:** 10.3390/jcm15124562

**Published:** 2026-06-12

**Authors:** Joon-Hyuk Lee, Il-Kug Kim, Sung-Eun Kim

**Affiliations:** Department of Plastic and Reconstructive Surgery, College of Medicine, Yeungnam University, Daegu 38541, Republic of Korea; mericle@naver.com (J.-H.L.);

**Keywords:** Behçet’s disease, oral squamous cell carcinoma, floor-of-mouth cancer, anterolateral thigh free flap, pectoralis major myocutaneous flap, wound dehiscence, pathergy, vasculitis, fistula, head and neck reconstruction

## Abstract

**Background/Objectives:** Behçet’s disease is a chronic relapsing multisystem inflammatory disorder characterized by recurrent mucocutaneous ulceration, vasculitis, and exaggerated inflammatory responses to minor trauma. These features may adversely affect wound healing after major head and neck oncologic reconstruction. This case report describes repeated wound breakdown after oral cavity reconstruction in a patient with Behçet’s disease and advanced floor-of-mouth squamous cell carcinoma. **Methods:** A 51-year-old woman with Behçet’s disease and T4N2bM0 squamous cell carcinoma involving the floor of the mouth and tongue underwent tumor resection followed by reconstruction of the oral cavity defect using a right anterolateral thigh perforator free flap. Subsequent surgical procedures included debridement of necrotic tissue, negative-pressure wound therapy, split-thickness skin grafting of the thigh donor site, and salvage tumor resection with pectoralis major myocutaneous flap reconstruction after tumor recurrence. **Results:** After the initial anterolateral thigh free flap reconstruction, flap perfusion was satisfactory in the immediate postoperative period; however, delayed marginal necrosis developed from the distal tongue-side flap margin, whereas the floor-of-mouth portion remained relatively stable. The right thigh donor site also developed progressive suture-line necrosis and wound dehiscence, requiring operative debridement, negative-pressure wound therapy, and split-thickness skin grafting. Although skin grafting achieved eventual donor-site coverage, partial graft necrosis and delayed secondary healing occurred. Persistent fistula and wound instability delayed postoperative radiotherapy, and recurrent floor-of-mouth squamous cell carcinoma subsequently developed approximately 6 months after the initial surgery. After salvage resection and pectoralis major myocutaneous flap reconstruction, the flap appeared viable at inset, but marginal ecchymosis, partial necrosis, and wound dehiscence again developed, requiring additional debridement, quilting sutures, and negative-pressure wound therapy. The wound gradually stabilized with staged wound management. **Conclusions:** This case illustrates a multifactorial pattern of repeated marginal wound breakdown after technically successful flap reconstruction in a patient with Behçet’s disease. Behçet-related pathergy-like inflammation, vasculitis, and microcirculatory dysfunction may represent possible contributing mechanisms, but they were not directly proven in this patient. In oral cavity reconstruction, such wound instability may delay adjuvant therapy and adversely affect oncologic outcomes. Careful perioperative planning, close multidisciplinary coordination, meticulous tension-free closure, early recognition of wound compromise, and readiness for staged wound management are essential in patients with Behçet’s disease undergoing major head and neck oncologic reconstruction.

## 1. Introduction

Oral cavity squamous cell carcinoma often requires wide surgical resection, and reconstruction is essential for restoring oral lining, separating the oral cavity from the neck, protecting exposed vital structures, and preserving swallowing and speech function. In tumors involving the floor of the mouth and tongue, the reconstructive strategy depends on defect size, tissue composition, tongue mobility, dead space, and the presence or absence of mandibular invasion. When mandibular invasion is present, mandibulectomy may require bony reconstruction. When the defect is predominantly soft tissue, fasciocutaneous free flaps such as the radial forearm free flap or anterolateral thigh flap are commonly used [1,2].

Behçet’s disease is a rare chronic multisystem inflammatory disorder characterized by recurrent oral ulceration, genital ulceration, ocular inflammation, skin lesions, and variable vascular, neurologic, gastrointestinal, and articular involvement [3,4]. Its pathophysiology involves complex interactions among genetic susceptibility, environmental triggers, immune dysregulation, neutrophil hyperreactivity, endothelial dysfunction, and vasculitis [3,4]. The pathergy phenomenon, in which minor trauma such as needle puncture or surgical incision induces an exaggerated inflammatory reaction, is particularly relevant in surgical patients [3].

From a reconstructive standpoint, Behçet’s disease may increase concern for excessive postoperative inflammation, incision-line ulceration, marginal necrosis, donor-site breakdown, and delayed epithelialization. Recent evidence suggests that postoperative complications in patients with Behçet’s disease vary according to surgical type, with surgical site complications and reoperations being clinically relevant concerns in selected procedures [5]. Earlier clinical observations also emphasized that wound healing in Behçet’s syndrome may be clinically unpredictable [6]. In the upper aerodigestive tract, Behçet-related pharyngeal or pharyngolaryngeal stenosis has required surgical intervention in selected cases, illustrating that mucosal inflammation and scarring can become clinically significant in surgically relevant head and neck regions [7,8,9]. In plastic surgery and wound reconstruction, case-based evidence has described delayed wound healing, superficial necrosis, or ulcer-related reconstructive challenges in patients with Behçet’s disease [10,11,12]. However, reports focusing specifically on oral cavity oncologic reconstruction in patients with Behçet’s disease remain limited.

Although the exact incidence or prevalence of oral cavity SCC arising in patients with Behçet’s disease has not been established due to a lack of large-scale statistical data, this co-occurrence remains an exceptionally rare clinical phenomenon, with only a limited number of cases documented worldwide.

Herein, we report a case of repeated wound breakdown after oral cavity reconstruction in a 51-year-old woman with Behçet’s disease and T4N2bM0 floor-of-mouth squamous cell carcinoma. The patient underwent initial anterolateral thigh free flap reconstruction, subsequent staged wound management, recurrence surgery, and salvage reconstruction using a pectoralis major myocutaneous flap. This case highlights the importance of considering Behçet’s disease as a possible contributing factor within a multifactorial wound-failure process, while also accounting for local wound conditions, perioperative medication planning, fistula prevention, and neck infection risk in reconstructive decision-making for complex oral cavity defects.

## 2. Case Presentation

### 2.1. Patient Information and Preoperative Assessment

A 51-year-old woman was diagnosed with squamous cell carcinoma involving the floor of the mouth and tongue. The cancer stage was T4N2bM0. She had no history of diabetes mellitus, was a non-smoker, and had no other significant systemic comorbidities apart from Behçet’s disease. Her nutritional status was not markedly compromised before surgery, with a body mass index of 21.5 kg/m^2^, serum albumin level of 4.1 g/dL, and prealbumin level of 24 mg/dL.

She had a known diagnosis of Behçet’s disease, which had been established at our institution by the Department of Rheumatology in 2009. Her preoperative Behçet’s Disease Current Activity Form (BDCAF) score was 1, suggesting clinically inactive or well-controlled disease activity at the time of surgery. Her regular medications for Behçet’s disease were colchicine 0.6 mg one tablet twice daily and methylprednisolone 1 mg one tablet twice daily. For the initial oral cavity cancer surgery and anterolateral thigh free flap reconstruction, both medications were stopped from one day before surgery until discharge according to the perioperative judgment of the rheumatology department, balancing infection risk, wound-healing concerns, and perioperative inflammatory disease control.

The case was reviewed preoperatively through multidisciplinary discussion involving otolaryngology-head and neck surgery, plastic and reconstructive surgery, rheumatology, and radiation oncology. Postoperative radiotherapy was planned as part of the oncologic treatment strategy but was later delayed and ultimately could not be initiated after the first operation because of persistent fistula and wound instability.

Preoperative evaluation emphasized the extent of tumor invasion, especially whether the mandible was involved. Mandibular invasion was considered an important determinant because mandibulectomy would have required bony reconstruction. For tongue and floor-of-mouth resection, soft-tissue reconstruction using either a radial forearm free flap or an anterolateral thigh free flap was considered. Based on the anticipated size and volume requirement of the defect, an anterolateral thigh perforator free flap was selected.

### 2.2. Initial Ablative Surgery and Anterolateral Thigh Free Flap Reconstruction

After wide excision of the malignant tumor by the otolaryngology-head and neck surgery team, a 14 × 8 cm open wound involving the oral cavity, floor of the mouth, and tongue was identified. Concurrently with the wide tumor excision, an ipsilateral right modified radical neck dissection encompassing Levels I to V was performed for the N+ neck, with preservation of the internal jugular vein, spinal accessory nerve, and sternocleidomastoid muscle. The preserved internal jugular vein and facial artery were subsequently used as recipient vessels for free flap microanastomosis. Copious normal saline irrigation was performed, and the wound bed appeared clean.

Recipient vessel exploration was performed through the open wound. The right facial arterial system was identified using Doppler ultrasonography and carefully dissected. A right anterolateral thigh flap measuring 14 × 8 cm was designed along a line connecting the anterior superior iliac spine and the superolateral border of the patella. Perforators were identified using Doppler ultrasonography, and flap elevation was performed in the subfascial plane while carefully preserving reliable perforators.

After confirming flap circulation, the flap pedicle was divided and the flap was transferred to the oral cavity defect. The flap was trimmed according to the wound shape. Under microscopic visualization, arterial microanastomosis was performed using 9-0 nylon between the perforator branch of the right lateral circumflex femoral artery system and the right facial arterial system in an end-to-side fashion. Venous microanastomosis was performed between the venae comitantes of the flap pedicle and the internal jugular vein. After revascularization, marginal bleeding and mild capillary refill were observed. The flap was inset, drains were placed, and the right thigh donor site was closed layer by layer.

No intraoperative or immediate postoperative complication was observed. The flap was pinkish, Doppler flow was intact, and mild capillary refill was confirmed immediately after surgery.

### 2.3. Delayed Oral Cavity Flap Necrosis After ALT Free Flap Reconstruction

**Figure 1:**
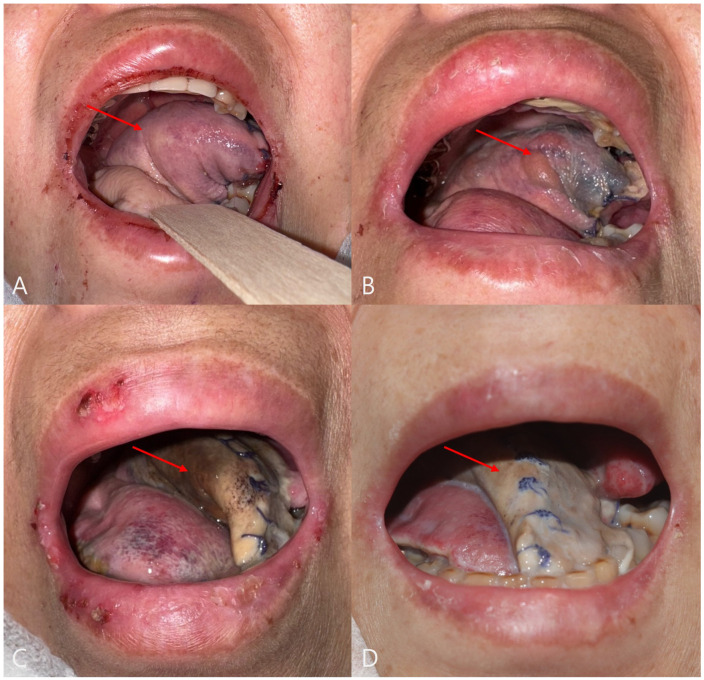
Serial postoperative findings of the oral cavity flap after anterolateral thigh free flap reconstruction. (**A**) POD 1, (**B**) POD 3, (**C**) POD 7, and (**D**) POD 14. The mouth-floor portion of the flap remained viable, whereas progressive necrosis developed from the distal margin of the tongue-side flap. By POD 14, partial flap necrosis was evident. The red arrows indicate the areas of marginal discoloration, progressive necrotic change, and wound dehiscence.

Although the flap initially appeared viable, delayed necrotic changes developed during postoperative follow-up. The mouth-floor portion of the flap remained relatively stable; however, necrosis gradually began at the distal margin of the tongue-side flap. By POD 14, partial flap necrosis was clearly observed. Because the reconstructed oral cavity communicated with the neck, fistula formation and secondary neck infection were important concerns during follow-up. The serial postoperative findings of the oral cavity flap after anterolateral thigh free flap reconstruction are shown in Figure 1.

The delayed pattern of necrosis was not consistent with immediate pedicle-level flap failure. However, this finding does not exclude routine flap-specific or local wound-related causes of marginal necrosis. The clinical course was interpreted as a multifactorial wound-failure process involving distal flap-edge perfusion limitations, salivary contamination, bacterial burden, tongue motion, local tension, repeated suture trauma, and the possible additional influence of Behçet’s disease-related inflammatory susceptibility.

### 2.4. Donor-Site Necrosis and Wound Dehiscence

**Figure 2:**
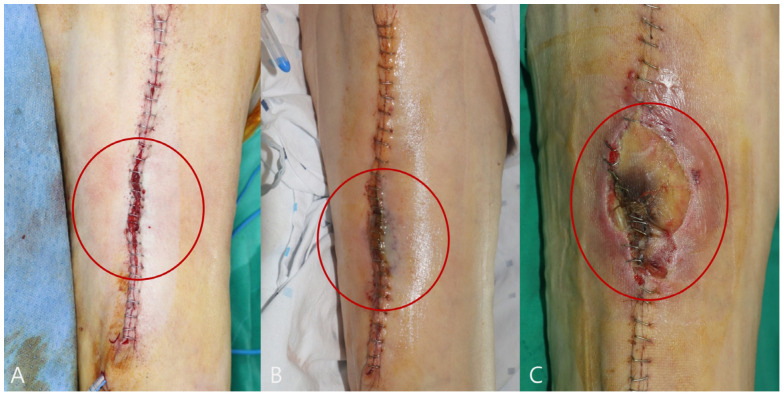
Serial postoperative findings of the right thigh donor site after anterolateral thigh flap harvest. (**A**) POD 1, (**B**) POD 3, and (**C**) POD 14. Necrosis began at the highest-tension portion of the suture margin and gradually progressed. By POD 14, wound dehiscence with extensive donor-site necrosis was evident. The red circles indicate the areas of progressive suture-line necrosis and donor-site wound dehiscence.

The right anterolateral thigh donor site also developed delayed wound deterioration. Necrosis began at the portion of the suture margin where closure tension appeared greatest. The necrotic change then progressed gradually along the donor-site incision, and by POD 14, extensive necrosis with wound dehiscence was observed. The serial postoperative findings of the right thigh donor site after anterolateral thigh flap harvest are shown in Figure 2.

The presence of donor-site necrosis was clinically important because it indicated that postoperative wound instability was not confined to the contaminated oral cavity recipient site. Rather, marginal breakdown occurred at both recipient and donor sites. This finding raised the possibility of systemic wound-healing vulnerability in addition to local mechanical and infectious factors, although a direct causal relationship with Behçet’s disease could not be proven.

### 2.5. Debridement, Skin Grafting, and Delayed Donor-Site Healing

The patient underwent operative debridement of necrotic tissue at the tongue-side flap margin, neck wound, and right anterolateral thigh donor site. Necrotic tissue was removed using electrocautery and sharp debridement. After bleeding control and copious saline irrigation, a closed suction drain was placed in the neck wound. The right thigh donor-site wound was debrided, and negative-pressure wound therapy was applied.

**Figure 3:**
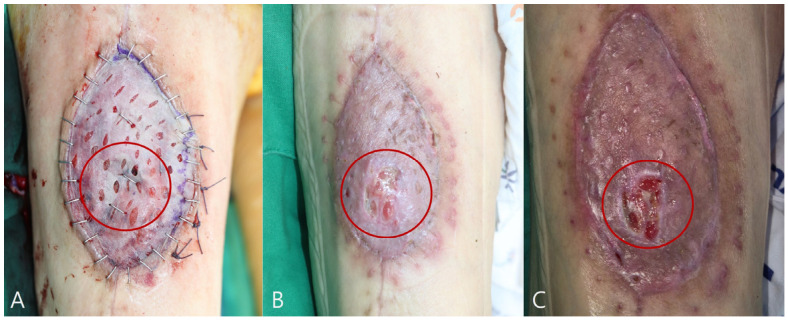
Serial postoperative findings after debridement and split-thickness skin grafting of the right thigh donor site. (**A**) POD 1, (**B**) POD 7, and (**C**) POD 1 month after skin grafting. The necrotic donor-site tissue was removed and covered with a split-thickness skin graft. A central portion of the skin graft underwent partial necrosis, and healing remained delayed until approximately one month after grafting. The residual wound was managed with outpatient ointment foam dressing and secondary healing. The red circles indicate the central area of partial graft necrosis and delayed wound healing.

Despite serial wound care, a persistent open wound remained at the right anterior thigh donor site. The necrotic tissue at the right thigh donor site was removed, and split-thickness skin grafting was performed for wound coverage. After skin grafting, the wound was followed serially. A central portion of the skin graft developed partial necrosis, and healing remained delayed until approximately one month after the procedure. The wound ultimately underwent secondary healing with outpatient ointment foam dressing. The serial postoperative findings after debridement and split-thickness skin grafting of the right thigh donor site are shown in Figure 3.

**Figure 4:**
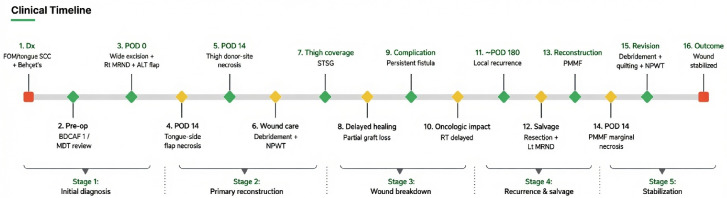
Summarized clinical timeline. The timeline shows the initial diagnosis, preoperative disease assessment, primary ablative surgery with right modified radical neck dissection and anterolateral thigh free flap reconstruction, postoperative oral cavity and donor-site wound breakdown, staged wound management, delayed adjuvant radiotherapy, local recurrence at approximately POD 180, salvage resection with left modified radical neck dissection, pectoralis major myocutaneous flap reconstruction, and subsequent wound stabilization.

After the first operation, postoperative radiotherapy could not be initiated because of persistent fistula and wound instability. Recurrent floor-of-mouth squamous cell carcinoma was identified approximately 6 months after the initial operation, and salvage tumor resection with pectoralis major myocutaneous flap reconstruction was subsequently performed at approximately POD 180 after the initial surgery. The overall clinical course is summarized in Figure 4.

### 2.6. Recurrent Floor-of-Mouth Squamous Cell Carcinoma and Salvage Reconstruction with a Pectoralis Major Myocutaneous Flap

At approximately POD 180 after the initial surgery, the patient developed recurrent squamous cell carcinoma of the floor of the mouth. She underwent salvage excision of the recurrent malignant tumor, left modified radical neck dissection, and tracheotomy by the otolaryngology-head and neck surgery team. After oncologic resection, a 7 × 5 cm open wound was present in the submandibular area.

At the time of salvage surgery and pectoralis major myocutaneous flap reconstruction, the patient continued medical treatment for Behçet’s disease with colchicine 0.6 mg one tablet twice daily and methylprednisolone 1 mg one tablet twice daily throughout the perioperative period. This change in medication strategy was influenced by the previous postoperative wound breakdown, persistent fistula, recurrent oncologic setting, and surgically altered neck. In this context, maintaining perioperative inflammatory disease control was considered clinically important, although the two reconstructive stages differed substantially in surgical and oncologic context.

Given the recurrent oncologic setting, previous free flap reconstruction, surgically altered neck, history of fistula, and repeated wound breakdown, salvage reconstruction was performed using a left pectoralis major myocutaneous flap.

The flap was designed using surface landmarks. A line was drawn from the left acromion to the xiphoid process, and a vertical reference line was marked between the mid-clavicle and distal third of the clavicle. The intersection of these lines was used to guide the superior border of the skin paddle. A second line from the manubrium to the humeral insertion of the pectoralis major was marked to identify the inferior border of the clavipectoral fascia. Doppler ultrasonography was used to trace the pectoral branch of the thoracoacromial artery.

After incision along the preoperative design, the flap was elevated in the submuscular plane while preserving the pectoral branch of the thoracoacromial artery. The pectoralis major muscle was dissected with maximal muscle sparing while retaining adequate tissue around the vascular pedicle. The flap was rotated around the thoracoacromial artery origin as the pivot point, and adequate pedicle length was confirmed. A 7 × 5 cm skin paddle was tunneled from the left chest donor site to the submandibular defect through a subfascial plane. Additional incision from the left clavicular area to the open wound was made to facilitate flap transfer. Pedicle integrity was reconfirmed with Doppler ultrasonography. The flap was inset into the submandibular defect using absorbable and nonabsorbable sutures, and closed suction drains were placed. The flap circulation appeared intact at the end of the operation.

### 2.7. Marginal Necrosis After PMMF Reconstruction

**Figure 5:**
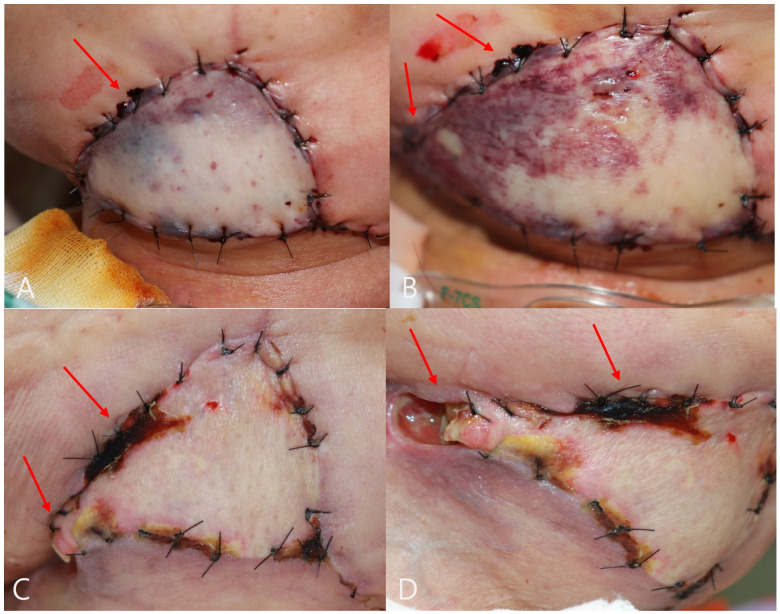
Serial postoperative findings after pectoralis major myocutaneous flap reconstruction. (**A**) POD 1, (**B**) POD 3, (**C**) POD 14, and (**D**) POD 14 lateral view. Ecchymotic change was noted at the flap margin from POD 1. By POD 14, wound dehiscence had developed, and necrotic tissue had formed a crust along the flap margin. The arrows indicate the areas of marginal ecchymotic change, necrotic crust formation, and wound dehiscence along the pectoralis major myocutaneous flap margin.

After pectoralis major myocutaneous flap reconstruction, ecchymotic change was noted at the flap margin from POD 1. By POD 7, partial marginal necrosis had developed, and by POD 14, wound dehiscence was observed with necrotic tissue forming a crust along the flap margin. The serial postoperative findings after pectoralis major myocutaneous flap reconstruction are shown in Figure 5.

The patient underwent operative debridement of the submandibular wound. Necrotic tissue and unhealthy granulation tissue were removed using a No. 15 blade and curette. After copious saline irrigation, the wound bed appeared clean. Quilting sutures were placed at the flap base to reduce dead space and stabilize the flap–wound interface. Negative-pressure wound therapy was then applied. With continued wound care, the submandibular wound gradually stabilized.

## 3. Discussion

This case demonstrates repeated wound breakdown after major oral cavity reconstruction in a patient with Behçet’s disease and T4N2bM0 floor-of-mouth squamous cell carcinoma. The most clinically relevant feature is that delayed marginal necrosis and dehiscence occurred repeatedly at multiple surgically traumatized sites, including the oral cavity flap margin, distal tongue-side flap margin, neck wound, right thigh donor site, skin graft site, and later the pectoralis major myocutaneous flap margin. However, the association between Behçet’s disease and wound failure in this patient should be interpreted cautiously because the evidence is circumstantial rather than definitive. The postoperative course was most likely multifactorial, involving extensive oncologic resection, oral contamination, local tension, fistula, repeated procedures, delayed adjuvant therapy, recurrent cancer, and possible systemic inflammatory susceptibility.

### 3.1. Oral Cavity Reconstruction, Fistula, and Neck Infection Risk

Reconstruction after floor-of-mouth and tongue cancer resection aims to restore oral lining, preserve tongue mobility, maintain swallowing and speech function, obliterate dead space, and separate the oral cavity from the neck. The radial forearm free flap and anterolateral thigh flap are widely used fasciocutaneous options for intraoral reconstruction. Ranganath et al. reported comparable outcomes between radial forearm and anterolateral thigh free flaps in oral cavity reconstruction, and Yang et al. also supported the anterolateral thigh flap as a useful option for large and complex head and neck defects [1,2].

Even in patients without systemic inflammatory disease, oral cavity wounds are prone to delayed healing because of saliva, bacterial contamination, tongue movement, dead space, and tension at the flap–mucosa interface. In floor-of-mouth reconstruction, wound breakdown may create communication between the oral cavity and neck, raising concern for fistula, secondary neck infection, delayed oral intake, and delay of adjuvant treatment. Therefore, these routine reconstructive risks must be considered before attributing marginal necrosis or wound dehiscence to Behçet’s disease.

In the present case, the immediate postoperative findings suggested successful revascularization, including pinkish flap color, intact Doppler signal, marginal bleeding, and mild capillary refill. Therefore, the subsequent necrotic change was unlikely to represent immediate pedicle-level thrombosis or technical microvascular failure alone. Nevertheless, the absence of immediate pedicle-level compromise does not exclude flap-specific or local causes of marginal necrosis, such as distal flap-edge perfusion limitations, local tension, salivary contamination, bacterial burden, tongue motion, dead space, and fistula formation.

### 3.2. Behçet’s Disease as a Possible Systemic Risk Factor for Wound Instability

Behçet’s disease is a chronic inflammatory disorder with mucocutaneous, ocular, vascular, neurologic, gastrointestinal, and articular manifestations [3,4]. From a reconstructive perspective, the disease is relevant because recurrent ulceration, neutrophil-driven inflammation, endothelial dysfunction, vasculitis, and pathergy-like tissue responses may theoretically affect wound repair after surgical trauma [3]. However, these mechanisms were not directly demonstrated in the present patient and should therefore be interpreted as possible explanations rather than disease-specific conclusions.

The patient’s preoperative BDCAF score was 1, suggesting clinically inactive or well-controlled Behçet’s disease at the time of surgery. Importantly, severe wound breakdown occurred despite this apparently inactive disease status. This observation suggests that even patients with well-controlled Behçet’s disease may remain vulnerable to pathergy-like wound responses after major reconstructive trauma, although this mechanism could not be directly proven in the present case.

Jung et al. reported postoperative complications in patients with Behçet’s disease, including wound dehiscence, bleeding, infection, anastomotic dehiscence, and stricture [5]. Mat et al. previously addressed wound healing in Behçet’s syndrome, reinforcing that postoperative wound behavior in this disease may be clinically unpredictable [6]. These studies support the clinical relevance of wound monitoring in Behçet’s disease, but they do not prove that Behçet’s disease was the direct cause of wound breakdown in the present case.

Reports involving other autoimmune or vasculitic disorders also provide useful context. A recent review of flap-based reconstruction in patients with autoimmune disease found that most studies did not demonstrate an independently increased risk of flap complications, whereas wound dehiscence and donor-site morbidity remained important concerns [13]. In contrast, Sanger et al. described a case of free flap anastomotic failure in which acute vasculitis was confirmed histologically [14]. These reports suggest that autoimmune or vasculitic conditions may influence reconstructive outcomes in selected patients, but extrapolation to Behçet’s disease and oral cavity free flap reconstruction should be made cautiously because direct evidence remains limited.

### 3.3. Hypothetical Mechanisms: Pathergy, Vasculitis, and Microcirculatory Dysfunction

The repeated marginal necrosis observed in this patient may be partly explained by hypothetical mechanisms related to Behçet’s disease, including pathergy-like inflammation, endothelial dysfunction, vasculitis, and impaired microcirculatory wound repair. Even when a flap has a patent axial pedicle or successful microvascular anastomosis, wound healing at the flap edge depends on local perfusion, neovascularization, fibroblast migration, collagen deposition, epithelialization, and resistance to bacterial contamination. These processes may be impaired at marginal zones exposed to tension, saliva, bacterial burden, and repeated suture trauma.

Coagulation and fibrinolytic abnormalities have also been reported in Behçet’s disease [15]. However, the present case did not show acute flap thrombosis, and no histopathologic examination of the wound edge was performed to confirm vasculitis or pathergy-related inflammatory change. Therefore, microvascular inflammatory dysfunction and thrombo-inflammatory changes should be considered hypothetical explanations rather than confirmed mechanisms in this patient.

### 3.4. Perioperative Medication Planning in Behçet’s Disease

The patient usually received colchicine 0.6 mg one tablet twice daily and methylprednisolone 1 mg one tablet twice daily for Behçet’s disease. For the initial oral cavity cancer surgery and anterolateral thigh free flap reconstruction, both medications were stopped from one day before surgery until discharge according to the perioperative judgment of the rheumatology department. This decision reflected a balance between infection risk, wound-healing concerns, and perioperative inflammatory disease control in the setting of major oncologic surgery and microvascular reconstruction.

Colchicine is commonly used for mucocutaneous and articular manifestations of Behçet’s disease and acts mainly by binding to intracellular tubulin and inhibiting microtubule polymerization, thereby suppressing neutrophil chemotaxis and inflammatory amplification [4,16]. Corticosteroids may also suppress inflammatory activity, although their influence on postoperative wound healing may vary depending on dose, duration, infection risk, nutritional status, and the overall surgical context.

In contrast, during the salvage surgery and pectoralis major myocutaneous flap reconstruction, colchicine 0.6 mg twice daily and methylprednisolone 1 mg twice daily were continued throughout the perioperative period. This change in strategy was influenced by the patient’s previous wound breakdown, persistent fistula, recurrent oncologic setting, and surgically altered neck. The different perioperative medication strategies should be interpreted cautiously: medication interruption during the initial operation cannot be assumed to have caused wound breakdown, and continuation of colchicine and low-dose methylprednisolone during the salvage PMMF reconstruction did not fully prevent marginal necrosis or wound dehiscence. Therefore, the wound course was most likely multifactorial, and perioperative inflammatory disease control should be individualized through multidisciplinary coordination.

### 3.5. Oncologic Consequence of Wound Breakdown and the Role of PMMF Salvage Reconstruction

A key oncologic consequence in this case was that postoperative radiotherapy could not be initiated after the first operation because of persistent fistula and wound instability. Recurrent floor-of-mouth squamous cell carcinoma was identified approximately 6 months after the initial surgery, and salvage tumor resection with left modified radical neck dissection, tracheotomy, and regional flap reconstruction was performed at approximately POD 180. This sequence emphasizes that wound complications after oral cavity cancer reconstruction should not be viewed only as local surgical morbidity; they may also interfere with timely adjuvant treatment and thereby complicate the overall oncologic course.

After recurrence, the pectoralis major myocutaneous flap was selected for salvage reconstruction in a previously operated and inflamed neck. Although free tissue transfer is now considered the standard for many complex head and neck defects, regional flaps remain important when a second free flap is undesirable or when the neck is vessel-depleted, hostile, previously dissected, irradiated, or medically high risk [17,18]. Liu et al. reported that pectoralis major myocutaneous flaps can be safely used in selected patients requiring salvage reconstruction, in patients at high risk for free flaps, and in patients requiring large-volume soft-tissue coverage [17]. Wei et al. also evaluated pectoralis major myocutaneous flaps in salvage reconstruction following free flap failure in head and neck cancer surgery, supporting their continued utility as a salvage option [18].

In the present case, PMMF reconstruction avoided additional microvascular anastomosis in a surgically altered neck and provided vascularized tissue for submandibular coverage. However, marginal necrosis and dehiscence again developed along the flap margin. This recurrence of marginal breakdown after a regional pedicled flap suggests that wound instability was not solely related to free flap microvascular factors. Instead, it likely reflected the combined effects of local wound conditions, repeated surgical trauma, recurrent oncologic disease, and possible systemic inflammatory susceptibility.

### 3.6. Limitations

This report has several limitations. First, it describes a single patient, and causality between Behçet’s disease and repeated wound breakdown cannot be proven. Second, the patient had multiple potential confounding factors, including extensive oral cavity oncologic surgery, salivary contamination, local tension, fistula, recurrent cancer, repeated operations, delayed adjuvant therapy, and a surgically altered neck. Third, although the patient had no diabetes mellitus, no smoking history, no other major systemic comorbidities, and no obvious nutritional compromise based on BMI, serum albumin, and prealbumin levels, these data do not exclude other local or systemic influences on wound healing. Fourth, although the preoperative BDCAF score was 1, indicating clinically inactive or well-controlled Behçet’s disease, detailed serial quantitative activity scores and inflammatory biomarker analyses were not available. Fifth, histopathologic examination of wound-edge vasculitis or pathergy-related inflammatory change was not performed. Therefore, Behçet’s disease should be interpreted as a possible contributing factor within a multifactorial wound-failure process rather than as a proven direct cause.

## 4. Conclusions

This case demonstrates repeated wound breakdown after oral cavity reconstruction in a 51-year-old woman with Behçet’s disease and T4N2bM0 floor-of-mouth squamous cell carcinoma. Although both the anterolateral thigh free flap and the pectoralis major myocutaneous flap appeared viable immediately after reconstruction, delayed marginal necrosis and dehiscence occurred repeatedly at the oral cavity flap margin, distal tongue-side flap margin, neck wound, thigh donor site, skin graft site, and PMMF margin. Persistent fistula and wound instability delayed postoperative radiotherapy, and recurrent cancer was identified approximately 6 months after the initial operation. Behçet’s disease may have increased susceptibility to postoperative wound instability through pathergy-like inflammation, vasculitis, endothelial dysfunction, and impaired microcirculatory wound repair; however, these mechanisms were not directly demonstrated in this patient. Therefore, the wound course should be interpreted as multifactorial. In patients with Behçet’s disease undergoing head and neck oncologic reconstruction, surgeons should anticipate possible delayed marginal wound complications, coordinate perioperative inflammatory disease control, minimize tissue trauma and tension, monitor closely for fistula and neck infection, and prepare for staged wound management when necessary.

## Data Availability

The data presented in this study are included in the article. Additional clinical information may be available from the corresponding author upon reasonable request, subject to institutional and ethical restrictions.

## Abbreviations

The following abbreviations are used in this manuscript:

ALT	Anterolateral thigh
NPWT	Negative-pressure wound therapy
POD	Postoperative day
PMMF	Pectoralis major myocutaneous flap
SCC	Squamous cell carcinoma
STSG	Split-thickness skin grafting
